# Post-transcriptional control of fungal cell wall synthesis

**DOI:** 10.1016/j.tcsw.2022.100074

**Published:** 2022-01-12

**Authors:** Rebecca A. Hall, Edward W.J. Wallace

**Affiliations:** a Kent Fungal Group, Division of Natural Sciences, School of Biosciences, University of Kent, Canterbury CT2 7NJ, United Kingdom; b Institute for Cell Biology and SynthSys, School of Biological Sciences, University of Edinburgh, EH9 3FF, United Kingdom

**Keywords:** RNA binding protein, Post transcriptional regulation, Cell wall synthesis, Fungi, Candida, RBP, RNA-binding protein, CWI, cell wall integrity pathway, RIP, RNA-immunoprecipitation, RIP-Chip, RIP followed by microarray (chip) analysis, CLIP-Seq, crosslinking and immunoprecipitation followed by sequencing, CRAC, UV cross-linking and high-throughput analysis of cDNAs, mRNP, messenger ribonucleoprotein (mRNA-protein) complex, RRM, RNA recognition motif protein domain

## Abstract

Pathogenic fungi hide from their hosts by camouflage, obscuring immunogenic cell wall components such as beta-glucan with innocuous coverings such as mannoproteins and alpha-glucan that are less readily recognised by the host. Attempts to understand how such processes are regulated have met with varying success. Typically studies focus on understanding the transcriptional response of fungi to either their reservoir environment or the host. However, such approaches do not fully address this research question, due to the layers of post-transcriptional and post-translational regulation that occur within a cell. Although in animals the impact of post-transcriptional and post-translational regulation has been well characterised, our knowledge of these processes in the fungal kingdom is more limited. Mutations in RNA-binding proteins, like Ssd1 and *Candida albicans* Slr1, affect cell wall composition and fungal virulence indicating that post-transcriptional regulation plays a key role in these processes. Here, we review the current state of knowledge of fungal post-transcriptional regulation, and link this to potential mechanisms of immune evasion by drawing on studies from model yeast and plant pathogenic fungi. We highlight several RNA-binding proteins that regulate cell wall synthesis and could be involved in local translation of cell wall components. Expanding our knowledge on post-transcriptional regulation in human fungal pathogens is essential to fully comprehend fungal virulence strategies and for the design of novel antifungal therapies.

## Introduction

The fungal cell wall is a dynamic multi-layered organelle composed of polysaccharides and proteins. The composition of the cell wall varies from species to species, but overall the cell wall consists of an inner skeletal layer of chitin and beta-glucan that forms the exoskeleton of the cell, maintaining cell shape, rigidity and turgor pressure, while the outer layer of the cell wall is formed of glycosylated mannoproteins, that provide specific cell functions like adhesion and invasion. For detailed information on specific cell structures we direct readers to the following review articles: (Orlean, 2012, Latgé et al., 2017, Patel and Free, 2019).

Multiple transcriptional pathways mount protective responses to cell wall stress. The cell wall integrity (CWI) pathway, along with other pathways like the *HOG1* and calcium-calcineurin pathway, is activated through membrane receptors, leading to a series of phosphorylation events activating Protein Kinase C (PKC) signalling, inducing the upregulation of a core set of cell wall biosynthesis genes (reviewed in (Dichtl et al., 2016, Rodríguez-Peña et al., 2010). One consequence of activation of the CWI pathway is the enhanced synthesis and incorporation of chitin in the cell wall, providing protection against environmental stress and resistance to antifungal treatment (Walker et al., 2008, Lee et al., 2012, Walker et al., 2013). Changes in the fungal cell surface affect the way the pathogen is perceived by the innate immune system. Indeed, the adaptation of *C. albicans* to host-specific environmental cues and antifungals affects exposure of the key immuno-stimulatory epitope beta-glucan, modulating immune recognition of the pathogen (Wheeler et al., 2008, Sherrington et al., 2017, Hopke et al., 2016, Ballou et al., 2016, Pradhan et al., 2019, Pradhan et al., 2018, Lopes et al., 2018). However, many of these cell wall perturbations appear not to be regulated by the CWI pathway.

In attempts to understand the molecular mechanism of differential beta-glucan exposure in *C. albicans*, the transcriptome of *C. albicans* has been analysed in response to lactate (Ballou et al. 2016), pH (Cottier et al. 2019), and mitochondrial respiration inhibitors (Duvenage et al. 2019). Although these approaches have identified genes that are differentially regulated under specific environmental conditions, to date they have not identified a core transcriptional signature associated with the exposure or concealment of beta-glucan. This suggests that, unlike the CWI pathway, cell wall remodelling leading to beta-glucan exposure may be controlled at levels other than transcriptional regulation. One caveat to this, is that these global transcriptional studies have been performed in different laboratories, using different media and growth conditions, on top of the different environmental signals under investigation, which might obscure transcriptional signatures related to cell wall remodeling. Accounting for such “batch effects” is critical in high-throughput studies (Leek et al. 2010).

Despite this lack of evidence for a core transcriptionally regulated response, proteomic analyses have identified differential expression of cell wall enzymes that regulate beta-glucan remodeling (Ene et al., 2012, Childers et al., 2020). This suggests a post-transcriptional regulatory module. Several well-established mechanisms lead to control of protein expression beyond a simple dependence on mRNA abundance (Dever et al., 2016, Verma‐Gaur and Traven, 2016). This means that transcriptome studies measuring only mRNA abundance cannot fully address the research question of which molecular mechanisms affect beta-glucan exposure. In agreement with this, cell wall proteomic studies in *C. albicans* identified differential protein abundances that are not predicted by differential mRNA abundances (Childers et al. 2020). However, the mechanisms of post-transcriptional regulation in pathogenic fungi have not been extensively studied. Here, we draw on knowledge from the model yeast, *S. cerevisiae,* and the plant pathogenic fungus, *Ustilago maydis,* regarding spatial and temporal post-transcriptional regulation to propose a model for how cell wall biosynthesis is regulated in pathogenic fungi and how this, in turn, contributes to immune recognition of human pathogenic fungi.

## Post-transcriptional regulation and cell wall synthesis

**Fig. 1:**
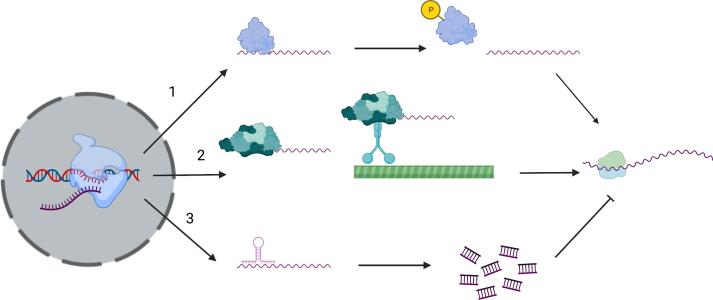
Mechanisms of post-transcriptional regulation. 1) RNA binding proteins (RBPs) interact with mRNAs inhibiting their translation, and phosphorylation of the RBP releases the mRNA, allowing translation. 2) RBPs bind mRNA, translationally silencing the mRNA, and the ribonucleoprotein complex is loaded onto motor proteins (i.e. Myosin 4, Dynein etc), which transport the mRNA complex along actin filaments or microtubules to its destination, where post-translational modification of the RBPs releases the mRNA and translation can occur. 3) Small non-coding RNAs base-pair to mRNA targeting its degradation by the RNA Induced Silencing Complex (RISC), inhibiting translation (Figure generated using Biorender).

Gene expression can be regulated at the transcriptional level through transcription factors and histone modifications, and post-transcriptionally by non-coding RNAs, upstream open reading frames, RNA binding proteins, mRNA localisation factors and mRNA decay machinery (Fig. 1). Global transcriptional approaches have provided a wealth of information on how genes involved in cell wall biogenesis are differentially regulated in response to different growth and stress conditions, but the role of post-transcriptional regulation is largely uncharacterised leading to a lack of understanding regarding the regulation of cell wall biosynthesis.

## Non-coding small RNAs

MicroRNAs or small interfering RNAs play key roles in post-transcriptional gene regulation. These 21–25 nucleotide RNAs base-pair to complementary mRNAs signaling their degradation by the RNA Induced Silencing complex (RISC) (Chang et al., 2012). *S. cerevisiae* lacks the machinery for RNA induced interference (RNAi), and microRNAs do not function in gene regulation in this model system (Drinnenberg et al. 2009). However, closely related Saccharomycete yeasts retain functional RNAi (Drinnenberg et al. 2009). RNAi systems have also been identified in other fungi including the thermal dimorphic fungal pathogens, as well as *Mucor circinelloides*, *Cryptococcus neoformans* and *Candida albicans* (Billmyre et al., 2013, Torres-Martínez and Ruiz-Vázquez, 2017). However, the role of this machinery in cell wall biosynthesis has not been extensively studied. In *Paracoccidioides brasiliensis* 49 microRNAs have been identified, with 44 being differentially regulated during morphogenesis, suggesting that microRNA-mediated post-transcriptional regulation is an important aspect of gene regulation during this switch (de Curcio et al. 2018). These microRNAs play key roles in repressing the expression of cell wall biosynthesis genes including hydrophobin, chitinase 3, endochitinase and glucan synthases during yeast phase growth (de Curcio et al. 2018). This post-transcriptional regulation mediated by microRNAs will be key to innate immune evasion of the fungus, as during infection *P. brasiliensis* yeast cells conceal beta-glucan from the immune system beneath an outer layer of alpha-glucan, promoting a Th2 response and continuation of infection (Souza et al. 2019).

Long non-coding RNAs (lncRNAs) in fungi are less well understood. We direct the interested reader to a review of lncRNAs and cell wall regulation in *S. cerevisiae* (Novačić et al. 2020), a recent bioinformatic analysis of lncRNAs in *Candida* species (Hovhannisyan and Gabaldón 2021), and a review of lncRNAs in fungi (Li et al. 2021).

## RNA degradation

Gene expression is also modulated via degradation of mRNA, which relies on a variety of abundant ribonucleases and accessory factors (Parker 2012). One major mRNA decay pathway conserved across eukaryotes is gated by deadenylation of the RNA by the Ccr4-Not complex (Panepinto et al., 2013). In most eukaryotes, this multi-subunit complex contains two active deadenylases, Ccr4 and and Pop2, that cooperate to shorten the poly(A) tail and release the poly(A) binding protein (Pab1) (Webster et al. 2018). However, in Saccharomycete yeasts the nuclease activity of Pop2/Caf1 has been lost (Panepinto et al., 2013). The Ccr4-Not complex also directly interacts with and ubiquitinates translating ribosomes (Collart 2016), and was recently shown in structural detail to monitor specific ribosomal states (Buschauer et al. 2020). Overall the roles of Ccr4-NOT in regulating gene expression are extensive and complex (Collart 2016).

Ccr4-NOT has been implicated in cell wall biogenesis and morphogenesis in several fungal species, reviewed in (Panepinto et al., 2013). In *S. cerevisiae*, Ccr4 regulates the cell wall integrity pathway by repressing the expression of *LRG1* mRNA (Duy et al., 2017). In. *C. albicans* the Ccr4-Not complex is involved in regulation of the cell wall integrity pathway, morphogenesis, virulence, mitochondrial function and phospholipid metabolism (Dagley et al. 2011). The deadenylation activity is crucial to these phenotypes, as shown by both deletion mutants and catalytically inactive mutants affecting morphogenesis and cell walls in thorough studies of both *S. cerevisiae* (Traven et al. 2009) and *C. albicans* (Dagley et al. 2011). The Ccr4-Not complex also affects the cell wall structure of *C. neoformans* (Bloom et al. 2019). Collectively, these results argue for a widely conserved role of mRNA deadenylation upstream of cell wall biosynthesis.

After the mRNA has been deadenylated, the mRNA cap is removed by the decapping enzymes Dcp1/Dcp2, and then mRNA is degraded by the exonuclease Xrn1 and the exosome complex (Parker 2012). Recently the RNA exosome complex has been shown to regulate cell wall integrity indirectly by maintaining correct protein glycosylation in response to stress (Novačić et al. 2021). Given that correct mannosylation of cell wall proteins is crucial for cell wall structure and concealment of beta-glucan (Hall and Gow 2013), dysregulation of the RNA exosome may contribute to immune recognition and evasion in pathogenic fungi.

## mRNA localisation

All organisms spatially regulate protein synthesis by transporting transcriptionally silent mRNA to sites of protein localisation. Four reasons have been proposed for this i) it promotes correct localisation of proteins ii) it prevents damaging mis-localisation of proteins, iii) transporting mRNA is potentially more energetically favourable than transporting individual proteins through the cell, and iv) it allows localised control of protein synthesis. Localised control is analogous to “just-in-time” manufacturing, where components are supplied only when and where they are needed.

**Fig. 2:**
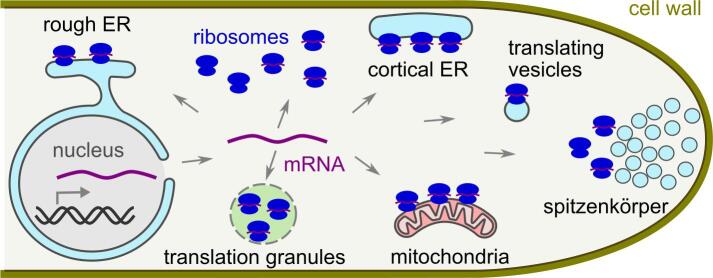
Locations of translation in fungal cells. Messenger RNA (purple) is transcribed in the nucleus, exported to the cytoplasm, and then translated by ribosomes (dark blue) in a variety of sub-cytoplasmic locations. We highlight the rough endoplasmic reticulum (ER) surface, cortical or plasma-membrane-proximal ER, translating vesicles, behind the spitzenkörper vesicle distribution center, the mitochondrial surface, and translation granules, as well as generic cytoplasmic ribosomes. Most ribosomes are shown complexed with mRNA, because 85% of ribosomes in fast-growing *S. cerevisiae* are engaged in active translation (Arava et al. 2003). (For interpretation of the references to color in this figure legend, the reader is referred to the web version of this article.)

Pioneering work by Grove and Bracker provided the first evidence that spatial and temporal regulation is important for cell wall biogenesis (Grove and Bracker 1970). The tips of fungal hyphae are packed with specialised vesicles, which contain the building machinery of the cell wall (i.e. glucan and chitin synthases) termed chitosomes (containing chitin synthases) and macrovesicles (containing glucan synthases) (Bartnicki-Garcia 2006). During active growth, these vesicles are secreted to the cell surface delivering the cell wall machinery to the plasma membrane. Located just behind these vesicles, are ribosomes (protein synthesis factories), mitochondria (power organelles) and endoplasmic reticulum (protein synthesis, modification, and trafficking sites) (Fig. 2) (Grove and Bracker 1970). Locating protein synthesis machinery in close proximity to the site of active growth could enable both correct localisation of cell wall components and “just-in-time” synthesis of the cell wall. This hypothesis is currently speculative, and it is unknown which mRNAs are enriched on tip-proximal ribosomes. However, any mRNAs near the growth tip must get there somehow, and this necessitates mRNA translocation.

### Transport of ribonucleoprotein complexes

The best-studied mechanisms of translocating mRNA through the cell require the use of RNA binding proteins (RBPs) that interact with motor proteins to move along the cytoskeleton. RNA binding proteins recognise and bind “zipcode” sequences within the mRNA, resulting in the recruitment of accessory proteins and formation of a messenger ribonucleoprotein (mRNP) complex (Niessing et al., 2018, Das et al., 2021). These transport mRNPs are then moved around the cell on actin filaments or microtubules through the actions of motor proteins.

One model system for mRNP transport involves the SWI5-dependent HO expression (SHE) protein complex comprised of She2 and She3 which transports *ASH1* to the emerging bud in *S. cerevisiae*, reviewed in (Niedner et al., 2014, Cosma, 2004). Translational repression of *ASH1* in the mother cell, and activation in the bud, leads to the Ash1 protein inhibiting mating-type switching in the daughter cell. To achieve this regulation, She2 is transported to the mother cell nucleus where it binds the *ASH1* mRNA. As the She2-mRNA complex exits the nucleus, additional RNA binding proteins (i.e. Puf6, Loc1, Khd1) associate with the complex, translationally inactivating the mRNA. In the cytoplasm, She2 then binds the mRNA-protein complex to She3 and Myo4, permitting transport of the complex along actin filaments. Once the mRNA complex is at the right subcellular location, the RBPs are phosphorylated releasing the mRNA, which can then be subsequently bound by ribosomes and translated into protein.

Moreover, the regulatory targets of the She pathway are species specific. *C. albicans* does not have an orthologue of She2, suggesting that either the mRNA-protein complex directly interacts with She3, or that this function is maintained through an as yet to be identified RNA binding protein. *C. albicans* has 2 orthologues of *SHE3* (Skrzypek et al. 2017), one of which is required for hyphal growth and for the transport of *ASH1* and other mRNAs to the hyphal tip (Elson et al. 2009). The mRNA targets of the She machinery in *C. albicans* include mRNAs that encode cell wall proteins like Cht2 (Elson et al. 2009), and the She complex is required for chitin remodelling in response to low pH (Sherrington et al. 2017). Therefore, at least in *C. albicans* the She complex is essential for correct cell wall biosynthesis.

The She pathway provides a paradigmatic example of protein localisation arising from active mRNA transport coupled to translation repression, relieved by localised translational activation. However, there are other RNA localisation systems in fungi that have different components and that might operate by somewhat different principles.

### Vesicles and endosomes as transporters of mRNA

Co-transport of mRNA with membrane-bound compartments, such as vesicles and endosomes, has emerged as an important principle, argued for in recent reviews (Haag et al., 2015, Béthune et al., 2019). Even She2 specifically interacts with areas of the cortical endoplasmic reticulum (Genz et al. 2013). In *U. maydis*, the Feldbrügge lab described a co-transport complex of RNA and endosomes involving specific RNA-binding proteins Rrm4 and co-factors, that are moved along microtubules (Baumann et al. 2014). Rrm4 regulates cell wall remodeling by binding the *CTS1* transcript that encodes a chitinase, and deletion of Rrm4 disrupts translation and secretion of the Cts1 protein (Koepke et al. 2011). Rrm4 is also required for hyphal extension, and ensures the transport of septin mRNAs for local translation, that in turn ensures correct septin cytoskeletal organization (Baumann et al. 2014). Analogously, in *C. albicans* hyphae the secretion regulator Sec2 is cotransported with *SEC2* mRNA and secretory vesicles (Caballero-Lima et al. 2014). “The common theme is that local translation supports the association of translation products with membranes, thereby guaranteeing their localization at the correct site,” as put by (Haag et al., 2015). This work raises questions as to the extent of transport of other mRNAs encoding polarity, secretion, and cell wall factors. For example, what other cell organisation proteins rely on localised translation for their localisation, and does this depend on co-transport of mRNA with membranes?

### Local translation as a more general principle

Surprisingly, recent work suggests that localised translation may be common even for proteins that are widely distributed within the cytoplasm (Fig. 2). Glycolytic enzymes, such as Pdc1 and Eno2, are translated in segregated sub-cytoplasmic granules in *S. cerevisiae* (Lui et al. 2014). Also in *S. cerevisiae*, several abundant translation factors are translated in separate sub-cytoplasmic granules, including at regions of polarized growth in a manner reminiscent of the hyphal spitzenkörper (Pizzinga et al. 2019).

These observations make a compelling case for investigating localised translation in fungi more generally, including morphologically complex species. Localising cell wall protein synthesis near the growth tip seems “obvious” given the localised requirement for these proteins, raising questions about which mechanisms might regulate such protein localisation. Currently, there is little direct evidence for localised translation of cell wall proteins near the growth tip. However, there is abundant evidence for translational control of cell wall proteins by specific RNA-binding proteins, which are themselves controlled by localised protein kinases.

## RNA-binding proteins

RNA binding proteins (RBPs) play essential roles in post-transcriptional regulation, including regulation of fungal cell wall synthesis. 21st-century methods have exploded our understanding of RBPs by identifying the RNA targets of many RBPs, and by identifying hundreds of new RBPs (Box 1). Currently, these methods have taught us a great deal about RNA-protein interactions in *S. cerevisiae*, humans, and some other model organisms including *U. maydis*, but we know very little about their diversification across fungi.

Box 1. Methods for measuring RNA-protein interactions These are reviewed in detail elsewhere (Ramanathan et al., 2019, Beckmann and Granneman, 2019). The pioneering method for globally identifying RNAs bound to a protein of interest was RNA-immunoprecipitation and microarray (RIP-Chip) (Keene et al., 2006). Here, antibodies are used to immunoprecipitate a protein of interest, together with its bound RNAs, from cell lysate; then microarrays measure the RNAs enriched in the immunoprecipitated sample compared to a control sample that is usually total mRNA. RIP-Chip and similar RIP-Seq methods suffer from false positives and false negatives due to changes in binding during cell lysis, a problem that is addressed by crosslinking RNA to protein within cells by UV light or chemicals (Riley and Steitz, 2013). Newer methods, CLIP-seq and CRAC, combine UV-crosslinking, immunoprecipitation, RNase digestion, and 2nd-generation sequencing to identify the precise sites of protein binding to RNA in living cells at nucleotide resolution (F. C. Y. Lee and Ule, 2018). Other methods identify RNA in close proximity to a protein of interest by fusing either an enzyme that edits RNA bases followed by sequencing (TRIBE; McMahon et al., 2016), or another that biotinylates nearby RNA (APEX-seq; Fazal et al., 2019, Padrón et al., 2019). Conversely, RNA interactome capture by crosslinking and proteomics methods allow measurement of all proteins bound to a set of RNAs. Crosslinked proteins are selected by binding to oligo(dT), which enriches for polyadenylated mRNA, or otherwise on silica columns that select for all RNA including ribosomal RNA, or by using organic solvents (Ramanathan et al., 2019). After digesting bound RNA, quantitative proteomics measures RNA-binding proteins that are reliably enriched in the RNA-crosslinked sample compared to a control sample of total protein. These data reveal a “brave new world” of thousands of RBPs, most of which do not contain canonical RNA-binding domains (Hentze et al., 2018). More precise proteomic analysis can also detect precise binding sites on the protein from mass “scars” on peptides that were crosslinked to RNA.

**Fig. 3:**
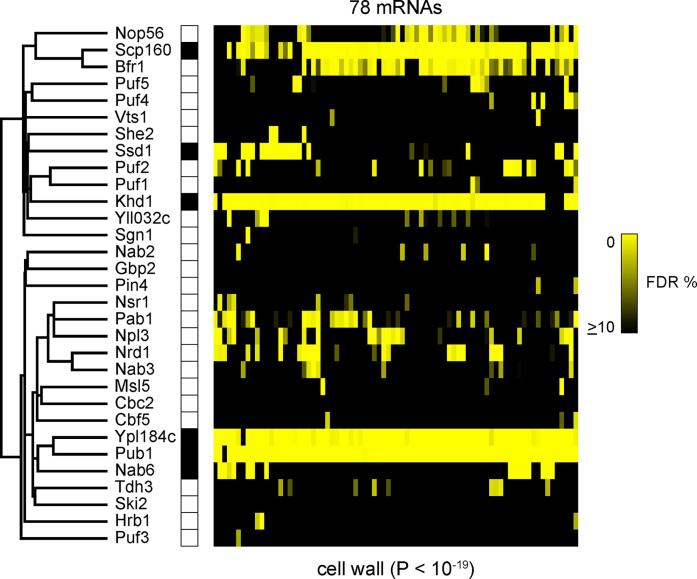
RNA-binding proteins co-operate to regulate fungal cell walls. RIP-Chip results of a subset of 78 mRNAs that associate with at least four of a set of six RBPs (Ssd1, Khd1/Hek2, Pub1, Mrn1/Ypl184c, Scp160, and Nab6), whose targets are enriched for mRNAs encoding proteins localized to the cell wall. See Table S1 for names of the mRNAs. “The heat map represents RBPs (rows) and mRNAs (columns) color coded to reflect the certainty with which we infer that a specific mRNA is a target of a specific RBP (10% FDR [black] to 0% FDR [yellow]).” This figure is reproduced from (D. J. Hogan et al. 2008)(CC-BY licence). (For interpretation of the references to color in this figure legend, the reader is referred to the web version of this article.)

In *S. cerevisiae*, a systematic RIP-Chip study of 42 predicted RNA-binding proteins confirmed that each protein bound mRNA, with some binding few mRNAs and some over a thousand different mRNAs (Hogan et al. 2008). From the 42 RBPs studied, seven were shown to bind mRNAs specifically enriched in cell wall synthesis (Ssd1, Scp160, Pub1, Mpt5, Mrn1, Khd1 and Bfr1), highlighting the importance of post-transcriptional regulation in *S. cerevisiae* cell wall biogenesis (Fig. 3). Cell wall proteins bound by at least 4 of these proteins include putative glucanases (*DSE2*, *SCW4*, *SCW10*, *SIM1*, *SUN4*, *UTH1*), an endochitinase (*CTS1*), mannoproteins (*PRY2*, *SED1*, *SRL1*, *TIR2*, *TOS1*), PIR repeat crosslinking proteins (PIR1, HSP150), and GPI-anchored proteins (*ECM33*, ​​*EGT2*, *FIT3*, *GAS3*, *GAS5*, *YNL190W*). Other regulators of the cell wall and cell wall protein glycosylation are also bound by these RBPs, as discussed by (Hogan et al. 2008); see Table S1 for a full list. Collectively these targets affect multiple aspects of cell wall structure, so that disruption of regulation by RBPs would have complex consequences.

While the study by Hogan *et al.* does not characterise all RNA binding proteins, this is the only study that has looked at yeast/fungal RNA binding proteins on such a large scale, allowing for direct comparisons between the mRNA targets. Further studies of this nature are required to fully appreciate the role post-transcriptional regulation plays in fungal biology, and particular attention should be placed on model pathogenic fungi if we want to understand the roles these regulatory proteins play in fungal virulence.

More recent crosslinking-based studies in *S. cerevisiae* mapped the precise binding sites of 13 (Tuck and Tollervey 2013) or 23 (Baejen et al. 2014) RNA-binding proteins, but did not focus on cell wall regulation. Targets of various other RBPs have been studied one at a time with a variety of methods, but it is difficult to draw precise comparisons between such varied datasets. Meanwhile, crosslinking and proteomics experiments collectively report over 500 new RBPs in *S. cerevisiae* (Beckmann et al., 2015, Mitchell et al., 2013). To date, the mRNA targets and regulatory functions of most of these protein-RNA interactions have not been studied in detail. In the following sections, the key RNA-binding proteins that are candidates to regulate the fungal cell wall are discussed. In most cases, these have been studied in *S. cerevisiae*, although even there the mechanistic links to cell wall regulation are unclear. With few exceptions, that we note, these RNA-binding proteins have not been studied in fungal pathogens.

### Ssd1

**Fig. 4:**
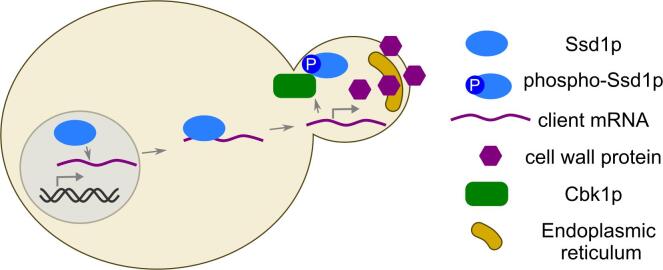
Model of post-transcriptional regulation by the RNA-binding protein Ssd1. Ssd1 binds mRNA co-transcriptionally in the nucleus, near the start codon, and represses translation. The repressed mRNA-protein complex travels or diffuses to sites of active growth, here the yeast bud. Localised Cbk1 kinase phosphorylates Ssd1, allowing translation of cell wall proteins from the mRNA. The proteins are then secreted through the ER to the cell wall. See text for references.

Ssd1 stands out as an important conserved regulator of fungal cell wall proteins. *S. cerevisiae* Ssd1 was discovered as a suppressor of deletion of *SIT4*, a protein phosphatase that regulates the cell cycle (Sutton et al., 1991). Ssd1 also appears as a hit in genetic screens for cell cycle, aneuploidy, temperature stress, and antifungal sensitivity (Wilson et al., 1991, Uesono et al., 1994, Mir et al., 2009, Hose et al., 2020, Schwarzmüller et al., 2014). Ssd1 homologues are required for virulence of diverse ascomycete fungal pathogens (Tanaka et al., 2007, Lee et al., 2010, Thammahong et al., 2019, Tanaka et al., 2009). This might be explained by Ssd1′s conserved role in regulating cell wall protein mRNAs (Hogan et al., 2008, Jansen et al., 2009, Nuñez et al., 2016, Herold et al., 2019, Bayne et al., 2021). Some of these cell wall targets encode glucanases and chitinases required for cell separation, and Ssd1 is also required for cell separation in “Titan” growth conditions of the distantly-related basidiomycete pathogen *Cryptococcus neoformans* (Ballou et al., 2021). Recently the precise Ssd1 binding sites have been identified in *S. cerevisiae*, and are located mostly near the start codons of mRNAs that encode a subset of cell wall proteins (Bayne et al. 2021). Deletion of *SSD1* promotes localisation and translation of some of these mRNA targets, which could explain the cell wall defects in *ssd1* mutant cells (Jansen et al., 2009, Kurischko et al., 2011a, Kurischko et al., 2011b). Ssd1 is thought to bind initially to nascent mRNA that is being transcribed in the nucleus (Kurischko et al., 2011b). Although the mechanism has yet to be directly established, it is hypothesised that Ssd1 directly negatively regulates protein synthesis by blocking ribosomes from accessing the start codon (Fig. 4). Many Ssd1 target mRNAs encode secreted proteins with signal peptides, and further work could investigate a link between Ssd1-dependent translation regulation and secretion of the protein product. Ssd1 could also have a role in environmental sensing because Ssd1 mutants are stress-sensitive (Mir et al., 2009), and the binding of Ssd1 to RNA is extraordinarily sensitive to stress (Bresson et al. 2020).

Ssd1 homologs also affect morphogenesis and RNA localisation in filamentous fungi. The *Neurospora crassa* homolog was identified in a morphogenetic screen for giant (“gulliver”) colonies and called Gul-1 (Terenzi and Reissig 1967). *N. crassa* and *Sordaria macrospora* Gul-1 move along hyphae in a microtubule-dependent manner, co-localised with endosomal markers (Herold et al., 2019, Stein et al., 2020). Localisation of Ssd1 in *S. cerevisiae* is less clear, as fluorescent fusions with Ssd1 at the native locus appear diffuse in the cytoplasm (Dubreuil et al. 2019), while overexpressed and/or mutant Ssd1-GFP fusions are found in cytoplasmic foci that may not represent native function (Kurischko et al., 2017, Kurischko et al., 2011b, Kurischko et al., 2011a). Further work is needed to understand the localisation of Ssd1 and its mRNA targets across the fungal kingdom.

Recent work argues that Ssd1′s RNA-binding activity is fungal-specific. Structurally, Ssd1 is a multi-domain protein related to the Dis3L2 family of RNases that is widespread in eukaryotes (Bayne et al. 2021). However, the nuclease activity of Ssd1 has been lost (Uesono et al., 1997), and a new RNA-binding site acquired in an ancestral fungus (Ballou et al., 2021, Bayne et al., 2021). We note that Ssd1 is not a protein phosphatase, although some homologues are erroneously listed as such in some databases (Basenko et al. 2018).

### Khd1

Khd1 (also called Hek2) binds several mRNAs that are located at the bud, that encode proteins that are located in the ER, cell wall and plasma membrane, in *S. cerevisiae* (Hogan et al., 2008, Hasegawa et al., 2008, Irie et al., 2002). Khd1 contains three K-homology RNA binding domains. While Khd1 is a translational repressor of She2/3 mRNA targets including *ASH1*, Khd1 can also act as an activator of gene expression for some transcripts including the plasma membrane localised stress sensor *MTL1* (Hasegawa et al., 2008). Khd1 binds (CNN)_n_ repeat elements in its target mRNAs, preventing the decapping enzymes from binding, and so increasing the stability of the mRNA (Hasegawa et al., 2008). A similar CNN-repeat element found in coding sequences of secreted proteins promotes protein secretion in a Khd1-dependent manner, revealing the potential of this mechanism for regulating the localisation of diverse targets (Cohen-Zontag et al. 2019).

Pbp2 is a homologue of Khd1 with similar domain structure, although Pbp2 has not been reported to regulate cell wall synthesis (Cherry et al. 2012). These proteins are both conserved in fungi, with the last common ancestor predicted early in fungal evolution (PTHR10288:SF309, (Mi et al. 2019)). Khd1 and Pbp2 are more distant homologs of eukaryotic poly(C)-binding proteins containing 3 K-homology- domains, named after mammalian heterogeneous nuclear ribonucleoprotein K (Nazarov et al., 2019, Makeyev and Liebhaber, 2002).

### Scp160 and Bfr1

Scp160 and Bfr1 are a pair of interacting RNA-binding proteins that regulate *S. cerevisiae* cell walls and directional growth. Scp160 is a direct effector of a signaling GTPase in the yeast mating pathway, and is required for mRNA localisation to the mating projection and for mating (Gelin-Licht et al. 2012). Scp160 is an orthologue of mammalian vigilin, an RNA-binding protein with multiple K-homology domains, the most C-terminal of which binds RNA, reviewed in (Cheng and Jansen 2017). Bfr1 is a predicted alpha-helix-rich protein that lacks a canonical RNA-binding domain (see AlphaFold prediction P38934 (Jumper et al. 2021)). Bfr1 is conserved in fungi, and has predicted homologues in nematodes and protists (PTHR31027, (Mi et al. 2019)), with similar predicted alpha-helix rich structures (Jumper et al. 2021). Confusingly, although *S. pombe* has a Bfr1 homolog, an unrelated gene in *S. pombe* is called *BFR1.*

Scp160 and Bfr1 bind an overlapping set of mRNAs enriched in cell wall proteins (Hogan et al. 2008), presumably at the same time as co-immunoprecipitation of Scp160 with Bfr1 is RNA-dependent (Lang et al. 2001). Both proteins are associated with polysomes at the endoplasmic reticulum surface, suggesting a role in translational control of secreted proteins (Frey et al., 2001, Lang et al., 2001). This polysome association depends on a specific ribosomal protein, Asc1, that can also act as an adaptor for other co-translational processes (Baum et al. 2004). Scp160, Bfr1, and the *S. pombe* vigilin homologue vgl1 all affect RNA localisation to the ER or cytoplasmic mRNA-protein granules, through unclear mechanisms (Weidner et al., 2014, Simpson et al., 2014, Wen et al., 2010). Indeed, *S. cerevisiae* Bfr1 is required for ER-localised translation of the ergosterol biosynthesis factor Erg4 (Manchalu et al. 2019). Bfr1 is also required for normal mannosylation, via translational regulation of ER-localised mannosyltransferases (Castells-Ballester et al. 2019).

The targets of Scp160 and Bfr1 are mostly distinct from those of Ssd1 in *S. cerevisiae*, which suggests a parallel pathway of translational control of secreted proteins (Fig. 3). Scp160 also operates independently of the She-actin system, because localisation of Scp160 targets does not depend on She2/3 (Gelin-Licht et al. 2012) and Scp160 association with the ER depends on microtubules, not actin (Frey et al., 2001). These proteins appear to have a second function in the nucleus, related to RNA-dependent gene silencing at heterochromatin and/or telomeres (Marsellach et al., 2006, Farooq et al., 2019). Unlike Ssd1, the precise sites of binding to mRNA are not known for Scp160 or Bfr1, although Scp160 appears to bind 3′UTRs of mating-tip-localised RNAs (Gelin-Licht et al. 2012).

### Pub1 and Mrn1

Pub1 is a highly abundant and conserved RNA-binding protein that is named for its binding of U-rich elements (Matunis et al., 1993, Anderson et al., 1993), and preferentially binds in 3′UTRs (Baejen et al. 2014). Pub1 was reported as enriched in binding to cell wall mRNAs and also ribosomal protein mRNAs in *S. cerevisiae*, both by Hogan *et al.* and by an independent RIP-Chip study (supplementary data of Duttagupta et al. 2005). Cell wall mRNAs bound by Pub1, such as *SED1*, may be regulated downstream of osmolarity pathways because they are differentially translated in a Hog1-dependent manner (Melamed et al., 2008). However, we are not aware of specific studies addressing Pub1′s role in cell wall biogenesis in fungi. Pub1, like the major poly(A)-binding protein Pab1, contains multiple RRM domains and an unstructured prion-like domain; in both proteins these domains cooperate to sense changes in cellular temperature and pH by triggering the formation of mRNP granules (Kroschwald et al., 2018, Riback et al., 2017). Pub1 is conserved beyond fungi (PTHR47640:SF5, Mi et al. 2019), with animal homologs TIA1/TIAR also binding U-rich sequences via their 3 RRMs (Dember et al. 1996), and having a functionally important prion-like domain (Su and Harrison, 2020, Li et al., 2014).

In *S. cerevisiae*, Mrn1 binding is associated with a U-rich element similar to that bound by Pub1 (Hogan et al. 2008), and it is possible that Mrn1 competes with Pub1 to bind shared mRNA targets (Reynaud et al. 2021). Mrn1, like Pub1, contains multiple RRM domains. The closest Mrn1 orthologue in *S. pombe*, Msa2/Nrd1, also binds U-rich sequences and has been studied due to its role as a negative regulator of sexual differentiation (Tsukahara et al., 1998, Oowatari et al., 2011). Confusingly, *S. cerevisiae* Nrd1 is an unrelated RNA-binding protein containing only 1 RRM (Cherry et al. 2012). Mrn1 is conserved throughout fungi and is part of a larger family of eukaryotic 4-RRM proteins including fungal splicing regulators Ecm2/Slt11 and Cwc2, and animal RBM22 (PTHR14089, Mi et al. 2019).

Experiments involving tethering of *S. cerevisiae* Mrn1 to reporter mRNAs found that Mrn1 binding can promote RNA decay. Mrn1 deletion increases the abundance of some of its targets shared with Pub1, such as mRNAs encoding the cell wall protein Sed1, and SUN-family proteins like Nca3. Reynaud *et al.* argue that Mrn1 integrates cell wall integrity and mitochondrial biosynthesis in a carbon-source responsive manner, particularly through Nca3 (Reynaud et al. 2021). Alternatively, overexpression of Mrn1 has also been reported to suppress mutations in chromatin remodeling factors and pre-mRNA splicing, hence the name “Multicopy suppressor of rsc nhp6” (Düring et al. 2012). The details of Mrn1 regulation of cell walls and morphogenesis through binding RNA remain to be resolved.

### PUF proteins

The PUF family of RNA binding proteins were initially discovered in *Drosophila melanogaster*, and are characterized by repeats of a distinctive Pumilio RNA-binding domain (Wang et al. 2002)*.* PUF family proteins are conserved throughout eukaryotes, and fungal PUF proteins are a model system for the evolution of RNA-binding proteins and their targets (Hogan et al., 2015, Lapointe et al., 2017). PUF proteins can promote RNA degradation by directly recruiting the CCR4-Not complex to RNAs (Goldstrohm et al., 2006, Webster et al., 2019). *S. cerevisiae* has 6 PUF family members (Puf1-6) which are involved in binding and regulating up to 10% of yeast mRNAs (Hogan et al., 2008, Quenault et al., 2011, Hogan et al., 2015, Gerber et al., 2004). Each of the *S. cerevisiae* PUF proteins functions in the regulation of mRNAs with a specific cellular localisation (Gerber et al., 2004). For example, Puf1-2 bind mRNAs that encode proteins located at the cell periphery, Puf3 binds mRNAs targeted to the mitochondria, and Puf4-5 bind mRNAs targeted to the nucleus and Puf6 represses *ASH1*. Although none of the PUF proteins are individually essential in *S. cerevisiae*, they are all regulated by environmental stress, again reinforcing the idea that post-transcriptional regulation is key for fungal adaptation and survival under stressful conditions.

Responses to cell wall stress are modulated by multiple PUF proteins in diverse fungi. In *S. cerevisiae*, deletion of either *PUF1/JSN1* or its paralog *PUF2* result in increased sensitivity to cell wall stress. These paralogous RNA binding proteins bind similar mRNA targets, and functionally compensate for each other. Deletion of *PUF1* or *PUF2* result in a mixture of increased and decreased abundance of target mRNAs, suggesting that these RBPs can act as post-transcriptional activators or repressors, depending on the target mRNA (Haramati et al. 2017). The effects of Puf1 and Puf2 on cell wall biosynthesis could be through their regulation of the cell wall integrity regulator Zeo1 (Haramati et al. 2017). Deletion of *PUF5*, also called *MPT5*, also increases sensitivity to cell wall stress in *S. cerevisiae* (Kaeberlein and Guarente 2002). Puf5 acts as an upstream activator of the cell wall integrity pathway by directly binding *LRG1* mRNA (Stewart et al., 2007, Viet et al., 2018).

In *C. neoformans*, Puf4 binds mRNA of *FKS1* and other cell wall associated genes stabilising the mRNAs, but repressing protein production (Kalem et al. 2021). Deletion of *PUF4* destabilises the mRNAs, resulting in translational activation, and increased protein expression of cell wall enzymes. The increased expression of these cell wall biosynthesis genes results in significant alteration to the structure and composition of the cell wall, including enhanced chitin incorporation, and so promoting echinocandin resistance (Kalem et al. 2021).

### Ddr48 and Cprp

Recent work identified a candidate family of conserved fungal RNA-binding proteins that could regulate the cell wall, orthologous to the FUS protein in humans (Mattos-Shipley et al., 2021). FUS is a mammalian RNA binding protein that regulates local translation (Fujii and Takumi, 2005, Yasuda et al., 2013), and that is characterised by a series of short GQSY repeats (Lagier-Tourenne and Cleveland 2009). Recently, a protein with repeats orthologous to FUS has been identified in the basidiomycete fungus *Clitopilus passeckerianus*, and deletion of this *Cprp* protein results in altered nitrogen metabolism and increased growth rate (Mattos-Shipley et al., 2021). The orthologue of Cprp in *S. cerevisiae* and *C. albicans* is Ddr48. In *S. cerevisiae*, Ddr48 has been identified in high throughput screens as an RNA binding protein (supplementary data of Shchepachev et al., 2019, Khoury et al., 2019). In *Clitopilus passeckerianus* Cprp is located at the cell wall and septum (Mattos-Shipley et al., 2021), suggesting that this protein could be linked to cell wall synthesis. Reports of the function of *C. albicans* Ddr48 are conflicting, with some suggesting that Ddr48 is an essential gene required for morphogenesis and stress responses (Dib et al. 2008), while other reports suggest that Ddr48 is dispensable for growth and morphogenesis, but plays a role in oxidative stress (Cleary et al. 2012). More importantly, deletion of *DDR48* resulted in increased resistance to cell wall stress (Cleary et al. 2012), suggesting that like other RNA binding proteins, Ddr48 might function as a regulator of cell wall biosynthesis.

### RBP interactions and dynamic regulation

Physical and genetic interaction data suggest that RNA binding proteins act together to regulate the cell wall: notably, their mRNA targets overlap (Hogan et al. 2008). However, the mechanisms of their cooperation are unclear, with one exception being recruitment of the CCR4-Not complex by PUF-family proteins. For example, Ssd1 co-operates with other RNA-binding proteins to regulate its targets, and double-deletions of *SSD1* and *PUF5/MPT5* are highly sensitive to cell wall stress (Kaeberlein and Guarente 2002). Similarly, double deletion of *CCR4* and *KHD1* has a severe cell lysis phenotype while the single gene deletions do not (Ito et al. 2011), suggesting functional overlap. Ssd1 and Pub1 also co-associate with other RBPs in a complex of specific size and unknown function (Zhang et al. 2014). Therefore, a wealth of information remains to be uncovered in relation to the combinatorial effects of RBPs on post-transcriptional regulation.

RNA binding of multiple cell wall co-regulators, including Ssd1 and Mrn1, is highly stress-sensitive (Bresson et al. 2020). Although the mechanisms behind this dynamic binding are not known in detail, phosphorylation of the RNA-binding proteins may play a role and connect to localised translational control. For example, Ssd1 is phosphorylated by the cell wall biogenesis kinase Cbk1 at a set of highly conserved phosphorylation sites on its N-terminus (Jansen et al. 2009). Cbk1 phosphorylation is thought to be the signal that releases Ssd1 from RNA and permits localised translation of the protein product (Fig. 4). This regulation is conserved across several fungal species. Cbk1 orthologues in *S. pombe* and *N. crassa* phosphorylate their respective Ssd1 orthologues, deletion of Ssd1 orthologues suppresses genetic defects from Cbk1 orthologue deletions, and Cbk1 target motifs are still more widely conserved in the N-termini of Ssd1 orthologues (Nuñez et al., 2016, Herold and Yarden, 2017, Ballou et al., 2021). Similarly, Khd1 is regulated via phosphorylation by a membrane-localised kinase, Yck1 (Paquin et al. 2007). Other RNA-binding proteins that we have discussed are also extensively phosphorylated near their probable RNA-binding sites (Y. Zhang and E. Wallace, unpublished analysis), but these have not been mechanistically investigated.

The phosphatases that counteract kinase regulation of these RBPs are mostly unknown. Phosphorylation of the Ssd1 homolog in filamentous fungi is regulated by the striatin-interacting phosphatase and kinase (STRIPAK) multi-subunit complex (Stein et al. 2020). The STRIPAK complex is known as the pheromone factor arrest (FAR) complex in *Saccharomyces* and is localised at the endoplasmic reticulum membrane (Kück et al., 2019, Kemp and Sprague, 2003, Pracheil and Liu, 2013), but we are aware of no reports linking the *Saccharomyces* complex to Ssd1 or other RBPs. Given that both FAR and Scp160 are involved in pheromone signaling and localised to the endoplasmic reticulum surface, it might be interesting to investigate FAR interactions with Scp160.

Overall, the evidence points to extensive post-transcriptional regulation of fungal cell walls by a set of interacting RNA-binding proteins regulated by (at least) protein kinases. This is conceptually consistent with the requirement for localised protein synthesis that many factors work together, as mRNAs in cells are constantly coated by many protein molecules (Singh et al. 2015). Different RNA-binding proteins could have distinct functions: restricting translation to one part of the cell requires some factors that directly facilitate transport, and other factors that inhibit protein synthesis outside the target location (Das et al. 2021).

## Role of post-translational regulation in immune recognition

Deletion of important regulatory units of cell wall biogenesis will result in aberrant cell wall synthesis, altered immune recognition, and altered virulence. Given that cell wall remodelling is required for fungal morphogenesis, deletion of RNA binding proteins involved in the regulation of cell wall biosynthesis results in morphological defects, attenuating virulence. At the same time, as with deletion of many of the glycotransferases, aberrant cell wall biosynthesis can lead to altered PAMP exposure increasing Dectin-1 dependent recognition of beta-glucan and promoting clearance of the pathogen (Hall and Gow 2013). For example, in *C. neoformans*, deletion of CCR4 leads to exposure of cell wall beta-glucan at host temperature and thus to immune recognition by the Dectin-1 receptor (Bloom et al. 2019). On the other hand, deletion of RNA binding proteins can have unexpected effects on the host-pathogen interaction through altered incorporation of cell wall proteins. For example, deletion of the RNA-binding protein *SLR1* increases *C. albicans* brain colonisation in murine infection models, which is hypothesised to be due to the enhanced expression of the fungal adhesin Als3 at the fungal cell surface (Ariyachet et al. 2013).

In environmental and clinical isolates of *S. cerevisiae*, deletion of *SSD1* increases the virulence in mouse infection models (Wheeler et al. 2003). This was attributed to changes in cell wall composition giving rise to a hyperactive immune response. On the other hand, deletion of *SSD1* in *C. albicans* attenuates virulence in murine infection models, attributed to increased sensitivity to antimicrobial peptides (Gank et al. 2008). In both yeasts, *ssd1* mutants are hypersensitive to cell wall perturbing agents like Congo red and Calcofluor white, suggesting that chitin synthesis is increased in the absence of functional Ssd1 (Kaeberlein and Guarente, 2002, Song et al., 2008). Under many conditions, elevated chitin levels also lead to increased beta-glucan exposure (Sherrington et al. 2017), which would enhance pro-inflammatory innate immune responses. On the other hand, in *A. fumigatus*, deletion of the *SSD1* homologue reduces sensitivity to Congo red and Calcofluor white, while its overexpression increases sensitivity to these cell wall stressors and also attenuates fungal virulence (Thammahong et al. 2019). We hypothesise that these complex effects arise from Ssd1-dependent regulation of multiple cell wall proteins and remodeling enzymes, as in *S. cerevisiae*. However, it is hard to extrapolate across highly diverged fungi with distinct cell wall structures. Therefore, it will be necessary to study the role of these RNA-binding proteins in multiple fungal pathogens to understand their effects on cell walls, and on immune recognition, in detail.

Given that many orthologues of the identified *S. cerevisiae* RNA binding proteins have not been studied in pathogenic fungi, it is likely that the importance of RNA binding proteins in controlling host-pathogen interactions and virulence is underestimated.

## Future perspectives

Recent developments in crosslinking and proteomic approaches indicate that approximately a sixth of genome-encoded proteins bind RNA (Hentze et al. 2018), most of which are likely to play roles in post-transcriptional gene regulation. To date, almost all published datasets on fungal RNA binding proteins come from *S. cerevisiae,* and currently there is limited knowledge of the roles orthologues of these regulatory proteins play in pathogenic or filamentous fungi. Given that filamentous fungi tend to have larger genomes than yeasts, we predict that these fungi will have distinctive RNA-binding proteins that are missing from *S. cerevisiae*. Therefore, currently there is a great underestimation of the role post-transcriptional regulation plays in cell wall biosynthesis and virulence in fungi.

### How do transcriptional and post-transcriptional regulation co-operate to regulate cell walls?

The distinct processes regulating gene expression must work together. In order for transcriptional induction of any cell wall integrity pathway to lead to cell wall repair, induced mRNAs must be translated into protein and then the proteins processed and correctly localised to the cell wall. When a cell wall integrity kinase is activated at sites of active growth, near to translationally stalled target mRNAs bound by RNA-binding proteins, it would seem strange for the kinase to bypass these and go straight to the nucleus to promote new transcription of identical mRNAs. Surely it would be faster and more efficient for these kinases to also promote translation at the growth site? For example, in *S. cerevisiae* the cell wall biogenesis kinase Cbk1 directly phosphorylates both the Ace2 transcription factor and the Ssd1 RNA-binding protein to co-ordinate cell separation (Colman-Lerner et al., 2001, Jansen et al., 2009). Ssd1 in turn binds mRNAs that are induced by Ace2, including mRNAs encoding a glucanase (Dse2) and a chitinase (Cts1), that together are secreted to degrade the cell septum. Future work should investigate co-operative regulation of transcription and post-transcriptional processes in cell wall remodelling, including by other kinases and phosphatases.

### How best to identify RNA binding proteins in pathogenic fungi?

Well-established methodologies for RNA interactome capture could be applied to discover the list of RNA-bound proteins in diverse fungi (Box 1). These have the advantage of not requiring any genetic engineering, instead relying only on UV crosslinking, standard molecular biology methods, and mass spectrometry proteomics.

### How to identify targets of RNA-binding proteins in pathogenic fungi?

Finding the mRNA targets of novel RBPs is more involved. Several RBP binding motifs have now been identified in *S. cerevisiae* and other model species. Therefore, it may be possible to identify post-transcriptionally regulated mRNAs through bioinformatic approaches, searching for model motifs in pathogen mRNA sequences. However, there are several limitations with this approach. First, pathogenic fungi, especially filamentous fungi, are likely to have RBPs that are not present in *S. cerevisiae* and therefore mRNAs regulated by these novel RBPs will not be identified in such an approach. Second, the binding motifs in highly divergent fungi could be diverged, leading to inaccurate estimation of target mRNAs. The PUF family provides such an example of diversification in RNA-binding proteins and their targets (Hogan et al., 2015). Third, sequence is an imperfect predictor of the actual sites of binding in living cells. Therefore, large scale CLIP-seq experiments will be best placed to identify RNA binding proteins and their mRNA targets in pathogenic fungi. For less well studied fungi like the Mucoromycota even this approach will be challenging, due to the incomplete genome annotation and reduced ability to genetically manipulate the fungi to express tagged proteins. Despite their limitations, new high-throughput studies will undoubtedly uncover novel RNA binding proteins, map their targets, and most likely provide new evidence for the roles of post-transcriptional regulation in fungal pathogenicity.

### How to find out the role of RNA-binding proteins in fungal growth and virulence?

Genetic screens can of course be used to identify RBPs that affect cell walls, growth, and virulence. This review argues that RBPs are as important to follow-up in such screens as transcription factors, protein kinases, and other “usual suspects”. Detailed follow-up by molecular genetics, cell biology, structural biology, and so on, is just as useful for RBPs as for other regulatory factors. In particular, characterising protein interaction partners will provide insights into the function and regulation of RBPs. Given that RBPs bind RNA and are often regulated by kinases, it is likely that designed mutations to RNA-binding domains and phosphorylation sites will be particularly informative, beyond searching for phenotypes of whole-gene deletions.

### How does post-transcriptional regulation contribute to environmental adaptation?

Fungi are highly adaptable and are able to grow in the presence of many environmental stimuli. Recent work on human pathogenic fungi has highlighted that the cell wall is highly dynamic with different environmental cues affecting its structure and composition (Hall 2015). To date the role of post-transcriptional regulation in environmentally induced cell wall remodelling has not been investigated extensively. Given that subsets of RBPs have been shown in several fungal species to bind mRNAs of key cell wall biosynthesis genes, it is highly likely that post-transcriptional regulation plays an important role here. In support of this hypothesis, beta-glucan synthases in *P. brasiliensis* are downregulated by microRNAs to promote beta-glucan masking (de Curcio et al. 2018). In *C. albicans,* several host derived environmental cues have been shown to induce glucan masking (Pradhan et al., 2018, Ballou et al., 2016, Pradhan et al., 2019, Lopes et al., 2018), and this masking involves trimming of exposed beta-glucan through the actions of exoglucanases similar to *P. brasiliensis* (Childers et al. 2020)*.* Therefore, it is likely that transcriptional regulation and post-transcriptional regulation co-operate to downregulate glucan synthases. In this same vein of thought, titan cell formation in *Cryptococcus* results in significant cell wall remodeling resulting in increased incorporation of glucosamine and mannose into the cell wall, but a decrease in the amount of glucose, suggestive of a decrease in glucan content (Mukaremera et al. 2018). Although the mechanisms behind this cell wall remodelling have not been investigated, it is possible that again answers will be found by studying post-transcriptional regulation.

In summary, there is wide scope for future work to explore functions of RNA-binding proteins and localised translation in fungi. It is likely that post-transcriptional regulation plays many roles in cell wall regulation, environmental responses, and host evasion, and that major discoveries await.

## CRediT authorship contribution statement

**Rebecca A. Hall:** Conceptualization, Writing – original draft, Writing – review & editing, Funding acquisition. **Edward W.J. Wallace:** Conceptualization, Writing – original draft, Writing – review & editing, Funding acquisition.

## Declaration of Competing Interest

The authors declare that they have no known competing financial interests or personal relationships that could have appeared to influence the work reported in this paper.
